# Supplementary material for: Evaluation of the current understanding of the impact of climate change on coral physiology after three decades of experimental research
Source: Commun Biol. 2022 Dec 26;5:1418. doi: 10.1038/s42003-022-04353-1 (PMC9792581; doi:10.1038/s42003-022-04353-1)
Supplement: Supplementary file 2 — Supplementary Information [file 42003_2022_4353_MOESM2_ESM.pdf]

Supplementary Materials for

Evaluation of the current understanding of the impact of climate change on coral physiology after three decades of experimental research

This PDF file includes:

Tables S1 to S7
Supplementary references for Table S1

Table S1: Summary of results derived from studies investigating the impact of ocean acidification on the physiology and algal and biochemical composition of tropical and temperate corals and anemones, together with the levels of pCO_2/pH and temperature used in the experiments.

Table S2: Two-way ANOVA tests analyzing direct and combined effects of ocean acidification and thermal-stress on maximum photochemical efficiency (F_v/F_m) at the end of the experiment.

Table S3: Statistical Student's t-tests in support of the differences found in the coral response to control condition over the experimental incubation.

Table S4: Two-way ANOVA tests analyzing direct and combined effects of ocean acidification and thermal-stress on photosynthesis, respiration and calcification rates, and the ratio of photosynthesis to respiration.

Table S5: Post hoc comparison using the Tukey HSD tests in support of the differences found in the coral response to the experimental treatments (heat-stress and ocean acidification).

Table S6: Results of the Principal Component Analysis (PCA) with the contribution of coral traits to principal component 1 (PC1) and 2 (PC2)

Table S7: Physicochemical conditions of the variation in temperature and pH over the course of the experiment for the four treatments.

Table S1.

Summary of results derived from studies investigating the impact of ocean acidification on the physiology (i.e., calcification, photosynthesis, respiration, photochemical efficiency) and algal and biochemical composition (i.e., symbiont and Chl *a* density, cellular *Symbiodinium* pigmentation C_i, protein content, coral tissue biomass, lipid and carbohydrate content) of tropical and temperate (khaki shaded) corals and anemones (blue shaded), together with the levels of pCO_2/pH and temperature used in the experiments. Studies have documented no effect (0), significant positive (+) or negative (−) effects of low-pH for different functional or structural coral traits. Studies performed on juvenile corals/larvae were not considered in this summary. Numbers refer to the way of experimental manipulation of the seawater carbonate chemistry applied in the study (^{1}Alkalinity/ Carbonate system manipulation,^{2}changing DIC at constant total alkalinity by bubbling with CO_2, ^{3}*in situ* incubations at low pH sites); duration refers to the length of each study (min = minutes, h = hours, d = days, w = weeks, m = months, yr = years); letters refer to the technique used for measuring calcification rates (^{A}Alkalinity anomaly, ^{B} Buoyant weight, ^{C} Linear Extension, ^{D} ^{45}Ca incorporation, ^{E}Cores, ^{F}Surface Area, ^{G}Cristal growth); symbols refer to different photosynthetic rates measured/calculated (* = Net photosynthesis, *^{C} = net carbon production, # =gross photosynthesis); ^{T} = Transcriptomic analysis, CSD = Cell Specific Density, HL = High Light, HT = High Temperature, LEDR = Light Enhanced Dark Respiration, LL = Low Light, LT = Low Temperature, NA = Not Available, NG = Not Given.

<

Study	Location	Genus species	Level	Duration	Temperature (°C)	Calcification	Photosynthesis	Respiration	Photochemical efficiency	Symbiont density	Chl*a* density	C_i	Protein content	Coral tissue biomass	Lipid content	Carbohydrate content
Agostini et al. 2013 ^{2}	Japan	*Galaxea fascicularis*	400 vs .750 ppm CO_2	5 d	27	NA	0 *	0	NA	NA	NA	NA	NA	NA	NA	NA
Anthony et al. 2008 ^{2}	Heron Island, Australia	*Acropora intermedia*	380 vs. 520–700 vs. 1000–1300 ppm CO_2	8 w	25-26	- ^{B}	- *	0	NA	NA	NA	NA	NA	NA	NA	NA
Anthony et al. 2008 ^{2}	Heron Island, Australia	*Porites lobata*	380 vs. 520–700 vs. 1000–1300 ppm CO_2	8 w	25-26	- ^{B}	- *	0	NA	NA	NA	NA	NA	NA	NA	NA
**Baghdasarian et al. 2017** ^{2}	Taiwan	*Seriatopora caliendrum*	472 µatm vs. 907 µatm CO_2	12 d	27.7	NA	NA	NA	NA	0	NG	0	0	NA	NA	NA
Bedwell-Ivers et al. 2016 ^{2}	Little Cayman Island	*Porites divaricata*	500 µatm vs. 1000 µatm CO_2	28 d	Ambient	- ^{B}/0 ^{C}	-*	-	NA	0	0	NG	NA	NA	NA	NA
Bedwell-Ivers et al. 2016 ^{2}	Little Cayman Island	*Aropora cervicornis*	500 µatm vs. 1000 µatm CO_2	28 d	Ambient	0 ^{BC}	0*	0	NA	0	0	NG	NA	NA	NA	NA
Camp et al. 2016 ^{1}	Little Cayman Island	*Acropora palmata*	Ambient vs. – pH 0.03 ± 0.02 units	35 d	Ambient	- ^{A}	- $^{\#}$	0	NA	-	-	NG	NA	NA	NA	NA
Camp et al. 2016 ^{1}	Little Cayman Island	*Porites astreoides*	Ambient vs. – pH 0.03 ± 0.02 units	35 d	Ambient	- ^{A}	- $^{\#}$	0	NA	0	0	NG	NA	NA	NA	NA
Castillo et al. 2014 ^{2}	USA	*Siderastrea siderea*	324 vs. 477 vs. 604 vs. 2553 µatm	95 d	28.1	parabolic ^{A}	NA	NA	NA	NA	NA	NA	NA	NA	NA	NA
Chauvin et al. 2011 ^{1}	La Reunion Island	*Acropora muricata*	1,440 – 340 µatm CO_2	2 h	27	- ^{A}	0 *	NA	NA	NA	NA	NA	NA	NA	NA	NA
Comeau et al. 2012 ^{1}	Moorea	*Porites rus*	280 – 2000 µatm CO_2	15 d	27.3	- ^{B}	NA	NA	NA	NA	NA	NA	NA	NA	NA	NA
Comeau et al. 2012 ^{1}	Moorea	*Porites rus*	280 – 2000 µatm CO_2	5-7 d	27.3	- ^{A}	NA	NA	NA	NA	NA	NA	NA	NA	NA	NA
Comeau et al. 2013 ^{2}	Moorea	*Porites rus*	280 – 2100 µatm CO_2 (pH 8.1 to pH 7.4)	2 w	27	- ^{B}	NA	NA	NA	NA	NA	NA	NA	NA	NA	NA
Comeau et al. 2013 ^{2}	Moorea	*Acropora pulchra*	280 – 2100 µatm CO_2 (pH 8.1 to pH 7.4)	2 w	27	- ^{B}	NA	NA	NA	NA	NA	NA	NA	NA	NA	NA
Comeau et al. 2013 ^{2}	Moorea	*Pocillopora damicornis*	280 – 2100 µatm CO_2 (pH 8.1 to pH7.4)	2 w	27	0 ^{B}	NA	NA	NA	NA	NA	NA	NA	NA	NA	NA
Comeau et al. 2013 ^{2}	Moorea	*Pavona cactus*	280 – 2100 µatm CO_2 (pH 8.1 to pH7.4)	2 w	27	- ^{B}	NA	NA	NA	NA	NA	NA	NA	NA	NA	NA
Comeau et al. 2014 ^{2}	Moorea	*Acropora pulchra*	400, 750 and 1100 µatm CO_2	20 d	27.2	- ^{B}	NA	NA	NA	NA	NA	NA	NA	+,-	NA	NA

Comeau et al. 2014 [2]	Moorea	*Porites rus*	280 – 2000 µatm CO_2	2 w	27	- [B]	NA	NA	NA	NA	NA	NA	NA	NA	NA	NA
Comeau et al. 2014[2]	Moorea	*Porites* spp.	280 – 2000 µatm CO_2	2 w	27	0 [B]	NA	NA	NA	NA	NA	NA	NA	NA	NA	NA
Comeau et al. 2014 [2]	Moorea	*Psammocora profundacella*	280 – 2000 µatm CO_2	2 w	27	- [B]	NA	NA	NA	NA	NA	NA	NA	NA	NA	NA
Comeau et al. 2014 [2]	Moorea	*Porites irregularis*	280 – 2000 µatm CO_2	2 w	27	- [B]	NA	NA	NA	NA	NA	NA	NA	NA	NA	NA
Comeau et al. 2014 [2]	Moorea	*Porites verrucosa*	280 – 2000 µatm CO_2	2 w	27	- [B]	NA	NA	NA	NA	NA	NA	NA	NA	NA	NA
Comeau et al. 2014 [2]	Moorea	*Pocillopora damicornis*	280 – 2000 µatm CO_2	2 w	27	0 [B]	NA	NA	NA	NA	NA	NA	NA	NA	NA	NA
Comeau et al. 2014 [2]	Moorea	*Acropora pulchra*	280 – 2000 µatm CO_2	2 w	27	- [B]	NA	NA	NA	NA	NA	NA	NA	NA	NA	NA
Comeau et al. 2014 [2]	Moorea	*Pavona cactus*	280 – 2000 µatm CO_2	2 w	27	- [B]	NA	NA	NA	NA	NA	NA	NA	NA	NA	NA
Comeau et al. 2014 [2]	Moorea, Hawaii, Japan	*Porites* spp.	400 vs. 700 vs. 1000 ppm CO_2	2 w	27	0 [B]	NA	NA	NA	NA	NA	NA	NA	NA	NA	NA
Comeau et al. 2014 [2]	Moorea, Hawaii, Japan	*Pocillopora damicornis*	400 vs. 700 vs. 1000 ppm CO_2	2 w	27	0 [B]	NA	NA	NA	NA	NA	NA	NA	NA	NA	NA
Comeau et al. 2017 [2]	Moorea	*Porites rus*	280 – 2000 µatm CO_2	7–10 d	27	NA	0*	0	NA	NA	NA	NA	NA	NA	NA	NA
Comeau et al. 2017 [2]	Moorea	*Porites* spp.	280 – 2000 µatm CO_2	7–10 d	27	NA	0*	0	NA	NA	NA	NA	NA	NA	NA	NA
Comeau et al. 2017 [2]	Moorea	*Psammocora profundacella*	280 – 2000 µatm CO_2	7–10 d	27	NA	0*	0	NA	NA	NA	NA	NA	NA	NA	NA
Comeau et al. 2017 [2]	Moorea	*Porites irregularis*	280 – 2000 µatm CO_2	7–10 d	27	NA	-*	0	NA	NA	NA	NA	NA	NA	NA	NA
Comeau et al. 2017 [2]	Moorea	*Pocillopora verrucosa*	280 – 2000 µatm CO_2	7–10 d	27	NA	0*	0	NA	NA	NA	NA	NA	NA	NA	NA
Comeau et al. 2017 [2]	Moorea	*Pocillopora damicornis*	280 – 2000 µatm CO_2	7–10 d	27	NA	0*	0	NA	NA	NA	NA	NA	NA	NA	NA
Comeau et al. 2017 [2]	Moorea	*Acropora pulchra*	280 – 2000 µatm CO_2	7–10 d	27	NA	0*	0	NA	NA	NA	NA	NA	NA	NA	NA
Comeau et al. 2017 [2]	Moorea	*Pavona cactus*	280 – 2000 µatm CO_2	7–10 d	27	NA	0*	0	NA	NA	NA	NA	NA	NA	NA	NA
Comeau et al. 2018 [1,2]	Western Australia	*Acropora yongei*	pH 8.24 vs. pH 8.01 vs. pH 7.83	13 w	20	- [B]	+*	NA	NA	NA	NA	NA	NA	NA	NA	NA
Comeau et al. 2018 [1,2]	Western Australia	*Pocillopora damicornis*	pH 8.24 vs. pH 8.01 vs. pH 7.83	8 w	20	0 [B]	NA	NA	NA	NA	NA	NA	NA	NA	NA	NA
Cornwall et al. 2018 [2]	Western Australia	*Goniapora* sp.	pH 8.05 vs. pH 7.65	100 d	26.5	0 [B]	0 [#]	0	NA	NA	NA	NA	NA	NA	NA	NA
Comeau et al. 2019 [2]	Western Australia	*Acropora yongei*	pH 8.05 vs. pH 7.65	27 w	20.5	- [B]	+*	NA	NA	NA	NA	NA	NA	NA	NA	NA
Comeau et a. 2019 [2]	Western Australia	*Plesiastrea versipora*	pH 8.05 vs. pH 7.65	27 w	20.5	- [B]	+*	NA	NA	NA	NA	NA	NA	NA	NA	NA
Crawley et al. 2010 [2]	Orpheus Island, Australia	*Acropora formosa*	Ambient vs. 600-790 vs. 1160-1500 ppm CO_2	4 d	23	NA	- *	+ LED R 0 Dark	NA	0	NG	+	NA	NA	NA	NA
Davies et al. 2016[2]	USA	*Siderastrea siderea*	324 vs. 477 vs. 604 vs. 2553 µatm	95 d	28.1	NA	NA	+ [T]	NA	NA	NA	NA	NA	NA	NA	NA
Diaz-Pulido et al. 2011 [2]	Australia	*Acropora intermedia*	400-1140 ppm CO_2	8 w	24-25	- [C]	NA	NA	NA	NA	NA	NA	NA	NA	NA	NA
Edmunds 2011 [2]	Moorea	*Porites* spp.	416 vs. 815 µatm CO_2	1 m	25.5	0 [B]	NA	NA	NA	0	NA	NA	NA	0	NA	NA
Edmunds 2012 [2]	Moorea	*Porites* spp.	429 vs. 766 vs. 872 µatm CO_2	11 d	28	0 [B]	NA	-	-	NA	NA	NA	NA	0	NA	NA
Edmunds et al. 2012 [2]	Moorea	*Porites rus*	416 vs. 815 µatm CO_2	1 m	25.6	- [B]	NA	NA	NA	NA	NA	NA	NA	NA	NA	NA
Edmunds et al. 2012 [2]	Moorea	*Porites* spp.	416 vs. 815 µatm CO_2	1 m	25.6	0 [B]	NA	NA	NA	NA	NA	NA	NA	NA	NA	NA
Enochs et al. 2014 [2]	Florida, USA	*Acropora cervicornis*	500 – 900 µatm CO_2	28 d	28	- [B]/0 [C]	NA	NA	0	NA	NA	NA	NA	NA	NA	NA
Fabricius et al. 2011[3]	Papa New Guinea	*Pocillopora damicornis*	pH 8.1 vs. pH 7.8	6 d	Ambient	- [C]	NA	NA	NA	NA	NA	NA	NA	NA	NA	NA
Fabricius et al. 2011 [3]	Papa New Guinea	*Porites* spp.	pH 8.1 vs. pH 7.8	NA	Ambient	0 [E]	NA	NA	NA	NA	NA	NA	NA	NA	NA	NA

Fine & Tchernov 2007 [2]	Mediterranean Sea	*Oculina patagonica*	pH 8.0-8.2 vs. pH 7.3-7.6	1 yr	Ambient (17-30°C)	- [B]	NA	NA	NA	NA	NA	NA	NA	NA	NA	NA
Fine & Tchernov 2007 [2]	Mediterranean Sea	*Madracis pharencis*	pH 8.0-8.2 vs. pH 7.3-7.6	1 yr	Ambient (17-30°C)	- [B]	NA	NA	NA	NA	NA	NA	NA	NA	NA	NA
Godinot et al. 2011 [2]	Red Sea	*Stylophora pistillata*	8.1, 7.8 and 7.5	10 d	26	NA	0 *	0	0	0	0	NG	0	NA	NA	NA
Hii et al. 2009 [2]	Malaysia	*Porites cylindrica*	pH 8.2 vs. pH 7.9	15 d	26	- [A]	- [*#]	-	NA	-	NG	0	NA	NA	NA	NA
Hii et al. 2009 [2]	Malaysia	*Galaxea fascicularis*	pH 8.2 vs. pH 7.9	15 d	26	+ [A]	0*/+ [#]	+	NA	-	NG	+	NA	NA	NA	NA
Hoadley et al. 2016 [2]	USA	*Montipora hirsuta*	400 vs. 800 µatm CO_2	18 d	26.5	NA	0 *	NA	0	0	NG	0	NA	NA	NA	NA
Hoadley et al. 2016 [2]	USA	*Pocillopora damicornis*	400 vs. 800 µatm CO_2	18 d	26.5	NA	0 *	NA	+ at LT	0	NG	0	NA	NA	NA	NA
Hoadley et al. 2016 [2]	USA	*Discosoma nummiforme*	400 vs. 800 µatm CO_2	18 d	26.5	NA	0 *	NA	+ at HT	0	NG	0	NA	NA	NA	NA
Holcomb et al. 2010 [2]	Woods Hole, Mass., USA	*Astrangia poculata*	ambient vs. 780 ppm	6 m	25.8	- [B]	NA	NA	NA	NA	NA	NA	NA	NA	NA	NA
Holcomb et al. 2012 [2]	Woods Hole, Mass., USA	*Astrangia poculata*	ambient vs. 790 ppm	4 m	24	- [B]	NA	NA	NA	NA	NA	NA	NA	NA	NA	NA
Horwitz & Fine. 2014 [2]	Red Sea	*Porites* sp.	pH 8.18 vs. pH 7.62 vs. pH 7.31	60 d	Ambient (21.6–24.3)	NA	NA	NA	NA	-	0	NA	NA	-	NA	NA
Horwitz & Fine. 2014 [2]	Red Sea	*Favia favus*	pH 8.18 vs. pH 7.62 vs. pH 7.31	60 d	Ambient (21.6–24.3)	NA	NA	NA	NA	-	0	NA	NA	-	NA	NA
Horwitz & Fine. 2014 [2]	Red Sea	*Acropora eurystoma*	pH 8.18 vs. pH 7.62 vs. pH 7.31	60 d	Ambient (21.6–24.3)	NA	NA	NA	NA	0	0	NA	NA	-	NA	NA
Horwitz & Fine. 2014 [2]	Red Sea	*Stylophora pistillata*	pH 8.18 vs. pH 7.62 vs. pH 7.31	60 d	Ambient (21.6–24.3)	NA	NA	NA	NA	-	-	NA	NA	0	NA	NA
Houlbreque et al. 2012 [2]	Red Sea	*Stylophora pistillata*	pH 8.1 vs. pH 7.8 vs. pH 7.5	5 w	26	0 [D]	0 *	0	0	0	0	NG	NA	NA	NA	NA
Iguchi et al. 2012 [2]	Sesoko Island, Japan	*Porites australiensis*	pH 8.05 vs. pH 7.6 vs. pH 7.4	8 w	27	- [B]	NA	NA	-	0	NG	0	NA	NA	NA	NA
Jokiel et al. 2008 [1]	Hawaii, USA	*Montipora capitata*	186 vs. 440 µatm CO_2	10 m	Ambient	- [BC]	NA	NA	NA	NA	NA	NA	NA	NA	NA	NA
Jury et al. 2010 [1]	Curaçao	*Madracis mirabilis*	pH 8.06 vs. pH 7.78 vs. pH 7.6	2 h	27-28	0 [A]	NA	NA	NA	NA	NA	NA	NA	NA	NA	NA
Kaniewska et al. 2012 [2]	Australia	*Acropora millepora*	pH 8.2 vs. pH 7.9 vs. pH 7.7	28 d	26	0 [B]	- [*#]	-	NA	-	NG	NG	NA	NA	NA	NA
Kavousi et al. 2015 [2]	Japan	*Acropora digitifera*	400 vs 1000 µatm CO_2	26	28	0 [B]	NA	NA	0	0	NG	0	0	NA	NA	NA
Kavousi et al. 2015 [2]	Japan	*Montipora digitata*	400 vs 1000 µatm CO_2	26	28	- [B]	NA	NA	-	0	NG	+	+	NA	NA	NA
Kavousi et al. 2015 [2]	Japan	*Porites cylindrica*	400 vs 1000 µatm CO_2	26	28	- [B]	NA	NA	0	0	NG	-	0	NA	NA	NA
Krief et al. 2010 [2]	Israel	*Porites sp.*	pH 8.09 vs. pH 7.49 vs. pH 7.19	6-14 m	25	NG [B]/- [F]	NA	NA	NA	-	NA	NA	+	NA	NA	NA
Krief et al. 2010 [2]	Israel	*Stylophora pistillata*	pH 8.09 vs. pH 7.49 vs. pH 7.19	6-14 m	25	- [B] /- [F]	NA	NA	NA	-	NA	+	+	NA	NA	NA
Krueger et al. 2017 [2]	Red Sea	*Stylophora pistillata*	pH 8.1 vs. pH 7.8	6 w	26.2	0 [A]	+ [#]	0	0	0	NG	-	0	NA	NA	0
Langdon & Atkinson 2005 [1]	Hawaii, USA	*P. compressa/M. capitata*	391 vs. 526 vs. 781 µatm CO_2	1.5 h	Ambient	- [A]	+[*C]	NA	NA	NA	NA	NA	NA	NA	NA	NA
Langdon & Atkinson 2005 [1]	Hawaii, USA	*P. compressa/M. capitata*	460 vs. 789 µatm CO_2	1.5 h	Ambient			NA	NA	NA	NA	NA	NA	NA	NA	NA
Langdon et al. 2018 [2]	Florida, USA	*Acropora cervicornis*	380 vs. 800 ppm CO_2	62 d	26	0 [C]	NA	NA	0	NA	NA	NA	NA	NA	NA	NA
Langdon et al. 2018 [2]	Florida, USA	*Orbicella faveolata*	380 vs. 800 ppm CO_2	62 d	26	- [C]	NA	NA	-	NA	NA	NA	NA	NA	NA	NA
Leclercq et al. 2002 [2]	Monaco	Coral community	411 vs. 647 vs. 918 µatm CO_2	4-12 w	26	- [A]	0 *	+	NA	NA	NA	NA	NA	NA	NA	NA
Marubini & Atkinson 1999 [1]	Hawaii, USA	*Porites compressa*	pH 8.0 vs. pH 7.2	5 w	NG	- [B]	NA	NA	NA	NA	NA	NA	NA	NA	NA	NA
Marubini et al. 2001 [1]	Hawaii, USA	*Porites compressa*	186 vs. 440 µatm CO_2	6 w	26.2	- [B]	NA	NA	NA	NA	NA	NA	NA	NA	NA	NA

Marubini et al. 2003 [1]	Monaco	*Acropora verweyi*	pH 8.1 vs. pH 7.7	8 d	26.5	- [B]	NA	NA	NA	NA	NA	NA	NA	NA	NA	NA
Marubini et al. 2003 [1]	Monaco	*Galaxea fascicularia*	pH 8.1 vs. pH 7.7	8 d	26.5	- [B]	NA	NA	NA	NA	NA	NA	NA	NA	NA	NA
Marubini et al. 2003 [1]	Monaco	*Pavona cactus*	pH 8.1 vs. pH 7.7	8 d	26.5	- [B]	NA	NA	NA	NA	NA	NA	NA	NA	NA	NA
Marubini et al. 2003 [1]	Monaco	*Turbinaria reniformis*	pH 8.1 vs. pH 7.7	8 d	26.5	- [B]	NA	NA	NA	NA	NA	NA	NA	NA	NA	NA
Marubini et al. 2008 [1]	Monaco	*Stylophora pistillata*	pH 8.2 vs. pH 8.0 vs. pH 7.6	8 d	26.5	- [B]	0 *	NA	NA	NA	0	NA	0	NA	NA	NA
Movilla et al. 2012 [2]	Spain	*Cladocora caespitosa*	Ambient vs. 800 ppm	92 d	20	- [B]	NA	NA	NA	0	NA	NA	NA	NA	NA	NA
Movilla et al. 2012 [2]	Spain	*Oculina patagonica*	Ambient vs. 800 ppm	92 d	20	- [B]	NA	NA	NA	0	NA	NA	NA	NA	NA	NA
Muehllehner & Edmunds 2008 [1]	Moorea	*Pocillopora meandrina*	Ambient vs. pH 7.8	2 w	27	- [B]	NA	NA	0	NA	NA	NA	NA	NA	NA	NA
Muehllehner & Edmunds 2008 [1]	Moorea	*Porites rus*	Ambient vs. pH 7.8	2 w	27	- [B]	NA	NA	0	NA	NA	NA	NA	NA	NA	NA
Noonan et al. 2015 [2]	Orpheus Island, Australia	*Seriatopora hystrix*	pH 7.95 vs. pH 7.8	3 w	28	NA	+ [#]	0	+	NA	+	NA	0	NA	NA	NA
Noonan et al. 2015 [2]	Orpheus Island, Australia	*Acropora millepora*	pH 7.95 vs. pH 7.8	3 w	28	NA	0 [#]	0	+	NA	0	NA	0	NA	NA	NA
Ogawa et al. 2013 [2]	Heron Island, Australia	*Acropora aspera*	Ambient vs. +70 µatm CO_2	14 d	29-30	NA	NA	NA	0	0	NG	0	NA	NA	NA	NA
Ohde & Hossain 2004 [1]	Japan	*Porites lutea*	pH 7.9 – pH 8.6	6 h	25	- [A]	NA	NA	NA	NA	NA	NA	NA	NA	NA	NA
Ohki et al. 2013 [2]	Japan	*Acropora digitifera*	< 300 vs. 400 vs. 600 vs. 800 vs. 1000µatm	6 w	27.1	- [B]	NA	NA	0	NA	NA	NA	NA	NA	NA	NA
Okazaki et al. 2013 [1]	Florida,,USA	*Siderastrea radians*	Ambient vs. –pH 0.1-0.2	Incubations of 90 min in situ at bimonthly intervals over a 2-yr period	Ambient	- [B]	0 *	NA	NA	NA	NA	NA	NA	NA	NA	NA
Okazaki et al. 2013 [1]	Florida,,USA	*Solenastrea hyades*	Ambient vs. –pH0.1-0.2	Incubations of 90 min in situ at bimonthly intervals over a 2-yr period	Ambient	- [B]	0 *	NA	NA	NA	NA	NA	NA	NA	NA	NA
Renegar & Riegl 2005 [2]	Florida,,USA	*Acropora cervicornis*	Ambient vs. ~700 to 800 µatm CO_2	4 w	25.3	- [B]	NA	NA	NA	NA	NA	NA	NA	NA	NA	NA
Reynaud et al. 2003 [2]	NG	*Stylophora pistillata*	460 vs. 760 µatm CO_2	5 w	25	0 [B]	- *	0	NA	+ CSD	0	NA	0	NA	NA	NA
Ries et al. 2009 [2]	North Carolina, USA	*Oculina arbuscula*	409 – 2856 ppm CO_2	60 d	25	- [B]	NA	NA	NA	NA	NA	NA	NA	NA	NA	NA
Ries et al. 2010 [2]	North Carolina, USA	*Oculina arbuscula*	409 – 2856 ppm CO_2	60 d	25	- [BC]	NA	NA	NA	NA	NA	NA	NA	NA	NA	NA
Rodolfo-Metalpa et al. 2010 [2]	Mediterranean Sea	*Cladocora caespitosa*	400 vs. 700 µatm CO_2	1 yr	Ambient	0 [B]	NG	NG	0	NA	NA	NA	NA	NA	NA	NA
Rodolfo-Metalpa et al. 2010 [2]	Mediterranean Sea	*Cladocora caespitosa*	400 vs 700 µatm CO_2 (summary)	1 m	Ambient	0 [A]	0 *[#]	0	NA	0	0	NG	0	NA	NA	NA
Rodolfo-Metalpa et al. 2010 [2]	Mediterranean Sea	*Cladocora caespitosa*	400 vs 700 µatm CO_2 (winter)	1 m	Ambient	0 [A]	0 *[#]	0	NA	+	+	NG	0	NA	NA	NA
Rodolfo-Metalpa et al. 2011 [3]	Italy	*Cladocora caespitosa*	pH 8.1 – pH 7.3	3 m	Ambient	- [B]	NA	NA	NA	NA	NA	NA	NA	NA	NA	NA
Rodolfo-Metalpa et al. 2011 [3]	Italy	*Cladocora caespitosa*	pH 8.1 – pH 7.4	3 m	Ambient	- [D]	NA	NA	NA	NA	NA	NA	NA	NA	NA	NA
Rodolfo-Metalpa et al. 2011 [3]	Italy	*Balanophyllia europaea*	pH 8.1 – pH 7.3	3 m	Ambient	- [B]	NA	NA	NA	NA	NA	NA	NA	NA	NA	NA
Rodolfo-Metalpa et al. 2011 [3]	Italy	*Balanophyllia europaea*	pH 8.1 – pH 7.4	3 m	Ambient	+ [D]	NA	NA	NA	NA	NA	NA	NA	NA	NA	NA
Schoepf et al. 2013 [2]	USA	*Acropora millepora*	382 vs. 607 vs. 741 µatm CO_2	3.5 w	26.5	- [B]	NA	NA	NA	0	-	NG	0	0	+	-
Schoepf et al. 2013 [2]	USA	*Montipora montasteriata*	382 vs. 607 vs. 741 µatm CO_2	3.5 w	26.5	0 [B]	NA	NA	NA	-	0	NG	0	0	0	0
Schoepf et al. 2013 [2]	USA	*Pocillopora damicornis*	382 vs. 607 vs. 741 µatm CO_2	3.5 w	26.5	0 [B]	NA	NA	NA	0	0	NG	0	0	+	0

Schoepf et al. 2013 [2]	USA	*Turbinaria reniformis*	382 vs. 607 vs. 741 µatm CO_2	3.5 w	26.5	0 [B]	NA	NA	NA	0	-	NG	0	0	0	0
Suggett et al. 2012 [3]	Italy	*Anemonia viridis*	pH 8.2 to pH 7.6	NA	21	NA	+ [#]	+	0	+	NA	NA	NA	NA	NA	NA
Suggett et. al. 2013 [2]	UK	*Acropora horrida*	380 vs. 720 µatm CO_2	5-6 w	26	- [A]	+ [#]	0	NA	NA	NA	NA	NA	NA	NA	NA
Suggett et. al. 2013 [2]	UK	*Porites cylindrica*	380 vs. 720 µatm CO_2	5-6 w	26	- [A]	+ [#]	0	NA	NA	NA	NA	NA	NA	NA	NA
Takahashi et al. 2013 [2]	Japan	*Acropora digifera*	343 vs. 744 vs. 2142 µatm CO_2	5 w	29	0 [AB]	0 *	0	0	0	NA	NA	NA	NA	NA	NA
Towanda & Thuesen 2012 [2]	USA	*Anthopleura elegantissima*	370 vs. 420 vs. 2310 µatm CO_2	6 w	12	NA	+ [#]	+	NA	+	0	NG	NG	NA	NA	NA
Tremblay et al. 2013 [2]	Monaco	*Stylophora pistillata*	pH 8.1 vs. pH 7.2	6 m	25	NA	- [#]	0	NA	-	-	NA	-	NA	NA	NA
Venn et al. 2013 [2]	Monaco	*Stylophora pistillata*	pH 8.0 to pH 7.2	2 m	25	- [FG]	NA	NA	NA	NA	NA	NA	NA	NA	NA	NA
Wall et al. 2014 [2]	Taiwan	*Seriatopora caliendrum*	451 vs. 851 µatm CO_2	14 d	27.7	NA	0 *	0	0	0	0	0	NA	NA	NA	NA
Wall et al. 2017 [2]	Hawaii, USA	*Pocillopora acuta* LL (7.5 mol m^{-2} d^{-1})	435 vs. 957 µatm CO_2	32 d	25	0 [B]	NA	NA	NA	0	0	0	0	0	-	0
Wall et al. 2017 [2]	Hawaii, USA	*Pocillopora acuta* HL (15.7 mol m^{-2} d^{-1})	435 vs. 957 µatm CO_2	32 d	25	0 [B]	NA	NA	NA	0	0	0	-	0	-	0

>

Table S2. Two-way ANOVA tests analyzing direct and combined effects of ocean acidification (887 µatm) and thermal-stress (+2°C) on maximum photochemical efficiency (F_v/F_m) at the end of the experiment on the four coral species: *Pseudodiploria strigosa*, *Orbicella faveolata*, *Montastraea cavernosa* and *O. annularis sp.*; significant values ($P < 0.05$) are in bold.

**Species**	**Factor**	**df**	**F**	***P***
P. strigosa	Temperature	1	128.043	**< 0.001**
	pH	1	9.298	**0.010**
	Temperature x pH	1	7.207	**0.020**
	Error	12		
O. faveolata	Temperature	1	72.638	**< 0.001**
	pH	1	0.368	0.555
	Temperature x pH	1	0.615	0.448
	Error	12		
M. cavernosa	Temperature	1	53.218	**< 0.001**
	pH	1	0.060	0.810
	Temperature x pH	1	0.319	0.583
	Error	12		
O. annularis	Temperature	1	17.547	**0.001**
	pH	1	0.242	0.632
	Temperature x pH	1	0.526	0.482
	Error	12		

Table S3. Welch t-test in support of the differences found in the coral response to control condition over the experimental incubation: Results of independent Student's t-test for testing the effects of tank incubations (Day 0 vs. Day 10) on the variability of the structural (Chorophyll *a* [Chl*a*] and symbiont density, symbiont cell pigmentation [Ci]) and physiological (Gross photosynthesis [P_{max}], post-illumination respiration [R_L], ratio of photosynthesis to respiration [P:R] and calcification [G_{max}]) coral traits of *Pseudodiploria strigosa*, *Orbicella faveolata*, *Montastraea cavernosa* and *Orbicella annularis*. Significant values ($p < 0.05$) are in bold.

Parameter	Species	Mean difference	SE	df	t-value	*p*
**Chl *a* density**	*P. strigosa*	5.464	11.625	6	0.470	0.655
**(mg Chl *a* m^{-2})**	*O. faveolata*	-23.419	17.091	4.414	-1.370	0.236
	M. cavernosa	10.103	25.317	6	0.399	0.704
	O. annularis	-19.620	17.719	6	-1.107	0.311
**Symbiont density**	*P. strigosa*	-0.396	0.316	3.732	-1.25	0.282
**(x 10^6 cells cm^{-2})**	*O. faveolata*	-0.685	0.358	6	-1.915	0.104
	M. cavernosa	0.0693	0.785	6	0.088	0.933
	O. annularis	-1.319	0.670	6	-1.969	0.97
**Cellular Chl*a* concentration C_i**	*P. strigosa*	0.260	0.448	5	0.579	0.588
**(pg Chl *a* cell^{-1})**	*O. faveolata*	0.036	0.302	3.708	0.120	0.911
	M. cavernosa	0.291	0.316	6	0.921	0.392
	O. annularis	1.031	0.231	6	4.457	**0.004**

**P_{max}**	*P. strigosa*	-0.075	0.154	6	-0.489	0.642
**(µmol O_2 cm^{-2} h^{-1})**	*O. faveolata*	0.471	0.203	6	2.321	0.059
	M. cavernosa	-0.249	0.234	6	-1.062	0.329
	O. annularis	-0.229	0.218	6	-1.049	-1.049
**R_L**	*P. strigosa*	0.114	0.073	4.249	1.553	0.191
**(µmol O_2 cm^{-2} h^{-1})**	*O. faveolata*	-0.327	0.173	6	-1.895	0.107
	M. cavernosa	0.153	0.059	3.796	2.607	0.063
	O. annularis	0.001	0.082	6	0.009	0.993
**P:R**	*P. strigosa*	0.593	0.374	6	1.585	0.164
	O. faveolata	-0.185	0.256	6	-0.722	0.498
	M. cavernosa	0.058	0.333	6	0.173	0.869
	O. annularis	-0.323	0.177	6	-1.817	0.060
**G_{max}**	*P. strigosa*	-0.062	0.047	5	-1.306	0.248
**(µmol $CaCO_3$ cm^{-2} h^{-1})**	*O. faveolata*	0.045	0.111	6	0.400	0.703
	M. cavernosa	0.093	0.078	5	1.201	0.283
	O. annularis	0.150	0.055	5	2.729	**0.021**

Table S4. Two-way ANOVA tests analyzing direct and combined effects of ocean acidification (887 µatm) and thermal-stress (+2°C) on photosynthesis, respiration and calcification rates, and the ratio of photosynthesis to respiration of *Pseudodiploria strigosa*, *Orbicella faveolata*, *Montastraea cavernosa* and *O. annularis*; significant values (p < 0.05) are in bold.

**Parameter**	**Species**	**Factor**	**df**	**F**	***p***
**Chl *a* density (mg Chl *a* m^{-2})**	*P. strigosa*	Temperature	1	60.027	**< 0.001**
		pH	1	0.475	0.505
		Temperature x pH	1	4.223	0.064
		Error	11		
	O. faveolata	Temperature	1	9.844	**0.009**
		pH	1	1.930	0.192
		Temperature x pH	1	3.832	0.076
		Error	11		
	M. cavernosa	Temperature	1	14.484	**0.003**
		pH	1	0.520	0.485
		Temperature x pH	1	0.201	0.662
		Error	12		

	O. annularis	Temperature	1	47.619	**< 0.001**
		pH	1	9.390	**0.011**
		Temperature x pH	1	1.520	0.243
		Error	11		
**Symbiont density ($x 10^6$ cells cm^{-2})**	*P. strigosa*	Temperature	1	30.540	**< 0.001**
		pH	1	4.068	0.069
		Temperature x pH	1	4.035	0.070
		Error	11		
	O. faveolata	Temperature	1	12.775	**0.004**
		pH	1	0.195	0.667
		Temperature x pH	1	0.003	0.957
		Error	12		
	M. cavernosa	Temperature	1	1.442	0.253
		pH	1	0.608	0.451

		Temperature x pH	1	0.113	0.742
		Error	12		
	O. annularis	Temperature	1	15.813	**0.002**
		pH	1	1.098	0.315
		Temperature x pH	1	0.090	0.770
		Error	12		
**Cellular Chl *a* concentration C_i (pg Chl *a* cell^{-1})**	*P. strigosa*	Temperature	1	3.506	0.086
		pH	1	0.270	0.613
		Temperature x pH	1	1.450	0.252
		Error	12		
	O. faveolata	Temperature	1	0.028	0.870
		pH	1	1.988	0.184
		Temperature x pH	1	2.341	0.152
		Error	12		

	M. cavernosa	Temperature	1	23.454	**< 0.001**
		pH	1	0.013	0.911
		Temperature x pH	1	0.089	0.770
		Error	12		
	O. annularis	Temperature	1	1.068	0.324
		pH	1	2.230	0.163
		Temperature x pH	1	0.377	0.552
		Error	12		
**P_{max} (μmol O_2 cm^{-2} h^{-1})**	*P. strigosa*	Temperature	1	171.138	**< 0.001**
		pH	1	13.438	**0.004**
		Temperature x pH	1	12.913	**0.004**
		Error	11		
	O. faveolata	Temperature	1	43.473	**< 0.001**
		pH	1	0.005	0.944

		Temperature x pH	1	1.229	0.289
		Error	12		
	M. cavernosa	Temperature	1	132.861	< **0.001**
		pH	1	0.155	0.701
		Temperature x pH	1	0.002	0.968
		Error	12		
	O. annularis	Temperature	1	95.859	< **0.001**
		pH	1	0.615	0.448
		Temperature x pH	1	0.590	0.457
		Error	12		
**R_L (µmol O_2 cm^{-2} h^{-1})**	*P. strigosa*	Temperature	1	4.590	0.055
		pH	1	2.750	0.125
		Temperature x pH	1	1.321	0.275
		Error	11		

	O. faveolata	Temperature	1	2.754	0.123
		pH	1	1.598	0.230
		Temperature x pH	1	0.160	0.697
		Error	12		
	M. cavernosa	Temperature	1	52.715	**< 0.001**
		pH	1	2.462	0.143
		Temperature x pH	1	1.487	0.246
		Error	12		
	O. annularis	Temperature	1	36.620	**< 0.001**
		pH	1	0.554	0.471
		Temperature x pH	1	1.440	0.253
		Error	12		
**P:R**	*P. strigosa*	Temperature	1	160.078	**< 0.001**
		pH	1	0.024	0.880

	Temperature x pH	1	0.263	0.617
	Error	12		
O. faveolata	Temperature	1	92.705	**< 0.001**
	pH	1	1.853	0.198
	Temperature x pH	1	0.785	0.393
	Error	12		
M. cavernosa	Temperature	1	104.702	**< 0.001**
	pH	1	0.780	0.395
	Temperature x pH	1	0.060	0.811
	Error	12		
O. annularis	Temperature	1	95.751	**< 0.001**
	pH	1	5.305	**0.040**
	Temperature x pH	1	0.207	0.657
	Error	12		

**G_{max} (μmol $CaCO_3$ cm^{-2} h^{-1})**	*P. strigosa*	Temperature	1	145.129	**< 0.001**
		pH	1	5.209	**0.043**
		Temperature x pH	1	0.088	0.773
		Error	11		
	O. faveolata	Temperature	1	14.188	**0.003**
		pH	1	2.327	0.155
		Temperature x pH	1	0.004	0.949
		Error	11		
	M. cavernosa	Temperature	1	47.196	**< 0.001**
		pH	1	1.896	0.194
		Temperature x pH	1	0.518	0.485
		Error	12		
	O. annularis	Temperature	1	49.905	**< 0.001**
		pH	1	1.978	0.190

Temperature x pH	1	9.302	**0.012**
Error	10		

Table S5. Post hoc comparison using the Tukey HSD tests in support of the differences found in the coral response to the experimental treatments (heat-stress and ocean acidification). Analyses were focused on the variability shown by the following coral traits of *Pseudodiploria strigosa*, *Orbicella faveolata*, *Montastraea cavernosa* and *Orbicella annularis:* A) structural: Chorophyll *a* [Chl*a*] and symbiont density, symbiont cell pigmentation [Ci]; and B) physiological: Gross photosynthesis [P_{max}], light-enhanced respiration [R_L], ratio of photosynthesis to respiration [P:R] and calcification [G_{max}]. Experimental treatments: CT-COA – ambient temperature-ambient pH; CT-OA – ambient temperature-low pH; HT-COA – high temperature-ambient pH; HT-OA – high temperature-low pH. Significant values ($p < 0.05$) are indicated in bold.

Parameter	Species	Comparison	Mean difference	SE	*p*	95 % CI Lower Bound	95 % CI Upper Bound
**Chl *a* density**	*P. strigosa*	CT-COA – CT-OA	-27.130	12.075	0.166	-62.979	8.720
**(mg Chl *a* m^{-2})**		CT-COA – HT-COA	25.107	12.075	0.214	-10.742	60.957
		CT-COA – HT-OA	31.130	12.075	0.097	-4.719	66.980
		CT-OA – HT-COA	52.237	12.075	**0.005**	16.388	88.087
		CT-OA – HT-OA	58.260	12.075	**0.002**	22.411	94.110
		HT-COA – HT-OA	6.023	12.075	0.958	-29.827	41.873
	O. faveolata	CT-COA – CT-OA	13.622	20.614	0.91	-47.579	74.823
		CT-COA – HT-COA	53.820	20.614	0.092	-7.381	115.021

		CT-COA – HT-OA	47.817	20.614	0.148	-13.384	109.018
		CT-OA – HT-COA	40.197	20.614	0.259	-21.004	101.398
		CT-OA – HT-OA	34.195	20.614	0.385	-27.007	95.396
		HT-COA – HT-OA	-6.003	20.614	0.991	-67.204	55.198
	M. cavernosa	CT-COA – CT-OA	12.451	15.054	0.841	-32.244	57.146
		CT-COA – HT-COA	45.290	15.054	**0.047**	0.595	89.985
		CT-COA – HT-OA	48.186	15.054	**0.033**	3.490	92.880
		CT-OA – HT-COA	32.839	15.054	0.184	-11.856	77.534
		CT-OA – HT-OA	35.735	15.054	0.136	-8.961	80.429
		HT-COA – HT-OA	2.895	15.054	0.997	-41.800	47.590
	O. annularis	CT-COA – CT-OA	20.801	13.623	0.453	-19.646	61.247
		CT-COA – HT-COA	50.807	13.623	**0.013**	10.360	91.254
		CT-COA – HT-OA	65.133	13.623	**0.002**	24.686	105.579
		CT-OA – HT-COA	30.007	13.623	0.178	-10.440	70.453
		CT-OA – HT-OA	44.332	13.623	**0.03**	3.885	84.779
		HT-COA – HT-OA	14.326	13.623	0.724	-26.121	54.772
**Symbiont density**	*P. strigosa*	CT-COA – CT-OA	-0.593	0.231	0.099	-1.278	0.093
**(x 10^6 cells cm^{-2})**		CT-COA – HT-COA	0.692	0.231	**0.048**	0.006	1.377
		CT-COA – HT-OA	0.690	0.231	**0.048**	0.004	1.376

	CT-OA – HT-COA	1.284	0.231	**<0.001**	0.598	1.970
	CT-OA – HT-OA	1.283	0.231	**<0.001**	0.597	1.968
	HT-COA – HT-OA	-0.001	0.231	1	-0.687	0.684
O. faveolata	CT-COA – CT-OA	-0.207	0.589	0.984	-1.956	1.542
	CT-COA – HT-COA	1.466	0.589	0.113	-0.283	3.215
	CT-COA – HT-OA	1.305	0.589	0.174	-0.444	3.054
	CT-OA – HT-COA	1.673	0.589	0.062	-0.076	3.422
	CT-OA – HT-OA	1.512	0.589	0.099	-0.237	3.261
	HT-COA – HT-OA	-0.161	0.589	0.993	-1.910	1.588
M. cavernosa	CT-COA – CT-OA	0.330	0.419	0.858	-0.913	1.573
	CT-COA – HT-COA	0.455	0.419	0.704	-0.788	1.698
	CT-COA – HT-OA	0.586	0.419	0.522	-0.657	1.829
	CT-OA – HT-COA	0.125	0.419	0.99	-1.118	1.368
	CT-OA – HT-OA	0.256	0.419	0.927	-0.987	1.499
	HT-COA – HT-OA	0.131	0.419	0.989	-1.112	1.374
O. annularis	CT-COA – CT-OA	0.590	0.619	0.778	-1.249	2.429
	CT-COA – HT-COA	1.873	0.619	**0.046**	0.034	3.712
	CT-COA – HT-OA	2.200	0.619	**0.018**	0.362	4.040
	CT-OA – HT-COA	1.283	0.619	0.217	-0.556	3.122

		CT-OA – HT-OA	1.611	0.619	0.094	-0.228	3.450
		HT-COA – HT-OA	0.328	0.619	0.95	-1.511	2.167
**Cellular Chl *a* concentration, C_i (pg Chl *a* cell^{-1})**	*P. strigosa*	CT-COA – CT-OA	-0.322	0.666	0.961	-2.299	1.654
		CT-COA – HT-COA	0.315	0.666	0.964	-1.662	2.291
		CT-COA – HT-OA	1.126	0.666	0.369	-0.850	3.103
		CT-OA – HT-COA	0.637	0.666	0.776	-1.340	2.613
		CT-OA – HT-OA	1.448	0.666	0.185	-0.528	3.425
		HT-COA – HT-OA	0.812	0.666	0.627	-1.165	2.788
	O. faveolata	CT-COA – CT-OA	0.706	0.340	0.215	-0.302	1.715
		CT-COA – HT-COA	0.408	0.340	0.638	-0.601	1.416
		CT-COA – HT-OA	0.379	0.340	0.687	-0.629	1.387
		CT-OA – HT-COA	-0.298	0.340	0.816	-1.307	0.710
		CT-OA – HT-OA	-0.327	0.340	0.772	-1.336	0.681
		HT-COA – HT-OA	-0.029	0.340	1	-1.037	0.980
	M. cavernosa	CT-COA – CT-OA	0.071	0.543	0.999	-1.542	1.684
		CT-COA – HT-COA	1.975	0.543	**0.016**	0.362	3.588
		CT-COA – HT-OA	1.817	0.543	**0.026**	0.204	3.430
		CT-OA – HT-COA	1.904	0.543	**0.02**	0.291	3.517
		CT-OA – HT-OA	1.746	0.543	**0.033**	0.133	3.359

		HT-COA – HT-OA	-0.158	0.543	0.991	-1.771	1.454
	O. annularis	CT-COA – CT-OA	0.240	0.370	0.914	-0.875	1.354
		CT-COA – HT-COA	0.114	0.400	0.991	-1.090	1.318
		CT-COA – HT-OA	0.689	0.370	0.299	-0.426	1.804
		CT-OA – HT-COA	-0.125	0.400	0.989	-1.329	1.079
		CT-OA – HT-OA	0.449	0.370	0.632	-0.666	1.564
		HT-COA – HT-OA	0.575	0.400	0.505	-0.630	1.779
**P_{max}**	*P. strigosa*	CT-COA – CT-OA	-0.384	0.189	0.23	-0.945	0.177
**(μmol O_2 cm^{-2} h^{-1})**		CT-COA – HT-COA	0.789	0.189	**0.006**	0.227	1.350
		CT-COA – HT-OA	0.783	0.189	**0.006**	0.221	1.344
		CT-OA – HT-COA	1.173	0.189	**<0.001**	0.611	1.734
		CT-OA – HT-OA	1.167	0.189	**<0.001**	0.605	1.728
		HT-COA – HT-OA	-0.006	0.189	1	-0.567	0.555
	O. faveolata	CT-COA – CT-OA	-0.182	0.218	0.837	-0.829	0.465
		CT-COA – HT-COA	0.845	0.218	**0.01**	0.198	1.492
		CT-COA – HT-OA	1.005	0.218	**0.003**	0.358	1.652
		CT-OA – HT-COA	1.027	0.218	**0.002**	0.380	1.674
		CT-OA – HT-OA	1.187	0.218	**<0.001**	0.540	1.834
		HT-COA – HT-OA	0.160	0.218	0.882	-0.487	0.807

	M. cavernosa	CT-COA – CT-OA	0.038	0.124	0.989	-0.329	0.405
		CT-COA – HT-COA	1.011	0.124	**<0.001**	0.644	1.378
		CT-COA – HT-OA	1.042	0.124	**<0.001**	0.675	1.409
		CT-OA – HT-COA	0.973	0.124	**<0.001**	0.606	1.340
		CT-OA – HT-OA	1.004	0.124	**<0.001**	0.637	1.371
		HT-COA – HT-OA	0.031	0.124	0.994	-0.336	0.398
	O. annularis	CT-COA – CT-OA	0.003	0.217	1	-0.640	0.645
		CT-COA – HT-COA	1.382	0.217	**<0.001**	0.739	2.024
		CT-COA – HT-OA	1.619	0.217	**<0.001**	0.976	2.262
		CT-OA – HT-COA	1.379	0.217	**<0.001**	0.736	2.022
		CT-OA – HT-OA	1.617	0.217	**<0.001**	0.974	2.260
		HT-COA – HT-OA	-0.238	0.217	0.697	-0.881	0.405
**R_L**	*P. strigosa*	CT-COA – CT-OA	0.160	0.135	0.649	-0.241	0.561
**(µmol O_2 cm^{-2} h^{-1})**		CT-COA – HT-COA	-0.090	0.135	0.908	-0.491	0.311
		CT-COA – HT-OA	-0.044	0.135	0.988	-0.445	0.357
		CT-OA – HT-COA	-0.250	0.135	0.3	-0.651	0.152
		CT-OA – HT-OA	-0.204	0.135	0.464	-0.605	0.198
		HT-COA – HT-OA	0.046	0.135	0.986	-0.355	0.447
	O. faveolata	CT-COA – CT-OA	0.101	0.086	0.652	-0.154	0.356

		CT-COA – HT-COA	-0.077	0.086	0.81	-0.331	0.178
		CT-COA – HT-OA	-0.024	0.086	0.992	-0.279	0.231
		CT-OA – HT-COA	-0.178	0.086	0.218	-0.432	0.077
		CT-OA – HT-OA	-0.125	0.086	0.491	-0.380	0.130
		HT-COA – HT-OA	0.053	0.086	0.926	-0.202	0.307
	M. cavernosa	CT-COA – CT-OA	0.012	0.047	0.994	-0.127	0.150
		CT-COA – HT-COA	-0.279	0.047	**<0.001**	-0.417	-0.141
		CT-COA – HT-OA	-0.187	0.047	**0.008**	-0.325	-0.049
		CT-OA – HT-COA	-0.291	0.047	**<0.001**	-0.429	-0.152
		CT-OA – HT-OA	-0.199	0.047	**0.005**	-0.337	-0.061
		HT-COA – HT-OA	0.092	0.047	0.251	-0.046	0.230
	O. annularis	CT-COA – CT-OA	0.105	0.076	0.537	-0.121	0.330
		CT-COA – HT-COA	-0.261	0.076	**0.022**	-0.486	-0.035
		CT-COA – HT-OA	-0.285	0.076	**0.013**	-0.511	-0.060
		CT-OA – HT-COA	-0.365	0.076	**0.002**	-0.591	-0.140
		CT-OA – HT-OA	-0.390	0.076	**0.001**	-0.615	-0.164
		HT-COA – HT-OA	-0.025	0.076	0.988	-0.250	0.201
**P:R**	*P. strigosa*	CT-COA – CT-OA	-0.098	0.207	0.964	-0.711	0.516
		CT-COA – HT-COA	1.775	0.207	**<0.001**	1.161	2.389

	CT-COA – HT-OA	1.828	0.207	**<0.001**	1.214	2.441
	CT-OA – HT-COA	1.873	0.207	**<0.001**	1.259	2.486
	CT-OA – HT-OA	1.925	0.207	**<0.001**	1.311	2.539
	HT-COA – HT-OA	0.053	0.207	0.994	-0.561	0.666
O. faveolata	CT-COA – CT-OA	0.073	0.216	0.986	-0.567	0.712
	CT-COA – HT-COA	1.333	0.216	**<0.001**	0.693	1.972
	CT-COA – HT-OA	1.675	0.216	**<0.001**	1.035	2.315
	CT-OA – HT-COA	1.26	0.216	**<0.001**	0.620	1.900
	CT-OA – HT-OA	1.603	0.216	**<0.001**	0.963	2.242
	HT-COA – HT-OA	0.343	0.216	0.42	-0.297	0.982
M. cavernosa	CT-COA – CT-OA	0.108	0.238	0.968	-0.600	0.815
	CT-COA – HT-COA	1.683	0.238	**<0.001**	0.975	2.390
	CT-COA – HT-OA	1.873	0.238	**<0.001**	1.165	2.580
	CT-OA – HT-COA	1.575	0.238	**<0.001**	0.868	2.282
	CT-OA – HT-OA	1.765	0.238	**<0.001**	1.058	2.472
	HT-COA – HT-OA	0.190	0.238	0.854	-0.517	0.897
O. annularis	CT-COA – CT-OA	0.315	0.241	0.576	-0.401	1.031
	CT-COA – HT-COA	1.590	0.241	**<0.001**	0.875	2.306
	CT-COA – HT-OA	2.060	0.241	**<0.001**	1.345	2.776

		CT-OA – HT-COA	1.275	0.241	**<0.001**	0.560	1.991
		CT-OA – HT-OA	1.745	0.241	**<0.001**	1.030	2.461
		HT-COA – HT-OA	0.470	0.241	0.259	-0.246	1.186
**G_{max}** (**µmol $CaCO_3$ cm^{-2} h^{-1}**)	*P. strigosa*	CT-COA – CT-OA	0.0784	0.045	0.342	-0.056	0.213
		CT-COA – HT-COA	0.375	0.041	**<0.001**	0.251	0.499
		CT-COA – HT-OA	0.436	0.041	**<0.001**	0.311	0.560
		CT-OA – HT-COA	0.297	0.045	**<0.001**	0.163	0.431
		CT-OA – HT-OA	0.357	0.045	**<0.001**	0.223	0.491
		HT-COA – HT-OA	0.060	0.041	0.49	-0.064	0.185
	O. faveolata	CT-COA – CT-OA	0.088	0.089	0.755	-0.179	0.356
		CT-COA – HT-COA	0.224	0.082	0.08	-0.023	0.472
		CT-COA – HT-OA	0.320	0.082	**0.011**	0.073	0.568
		CT-OA – HT-COA	0.136	0.089	0.455	-0.132	0.403
		CT-OA – HT-OA	0.232	0.089	0.096	-0.035	0.499
		HT-COA – HT-OA	0.096	0.082	0.656	-0.151	0.344
	M. cavernosa	CT-COA – CT-OA	-0.036	0.078	0.965	-0.268	0.195
		CT-COA – HT-COA	0.419	0.078	**<0.001**	0.187	0.650
		CT-COA – HT-OA	0.303	0.078	**0.01**	0.071	0.535
		CT-OA – HT-COA	0.455	0.078	**<0.001**	0.223	0.686

	CT-OA – HT-OA	0.339	0.078	**0.005**	0.108	0.571
	HT-COA – HT-OA	-0.116	0.078	0.477	-0.347	0.116
O. annularis	CT-COA – CT-OA	-0.017	0.089	0.997	-0.284	0.249
	CT-COA – HT-COA	0.208	0.089	0.145	-0.058	0.475
	CT-COA – HT-OA	0.439	0.089	**0.002**	0.173	0.706
	CT-OA – HT-COA	0.225	0.082	0.077	-0.021	0.472
	CT-OA – HT-OA	0.457	0.082	**<0.001**	0.210	0.703
	HT-COA – HT-OA	0.231	0.082	0.069	-0.016	0.478

Table S6. Results of the Principal Component Analysis (PCA) with the contribution of coral traits to principal component 1 (PC1) and 2 (PC2)

**Coral Trait**	PC1	PC2
Symbiont density	-0.4171	-0.214
Chla density	-0.4203	0.1276
Symbionts Ci	-0.3069	0.4833
Calcification rate (G_{max})	0.0974	0.8115
Respiration rate (R_L)	-0.4151	0.1246
Photosynthesis rate (P_{max})	-0.4647	-0.0391
P/R ratio	-0.3967	-0.1694

Table S7. Physicochemical conditions of the variation in temperature and pH over the course of the experiment for the four treatments. Temperature (°C) and pH (on the NBS scale) at each experimental condition (CT-COA – Ambient temperature-ambient pH; CT-OA – Ambient temperature-low pH; HT-COA – high temperature-ambient pH; HT-OA – High temperature-low pH) represent averages of daily temperature and pH (*n* = 13) ± SE. Mean (± SE; *n* = 8) seawater carbonate chemistry (pCO_2, HCO_3^-, CO_3^{2-} and Ω_{arag}) for the various experimental treatments were derived from measurements of pH and Total alkalinity (TA) of water samples using the software CO2SYS[71].

Treatment	Temperature (°C)	pH_{NBS}	TA (µlmol kg^{-1})	pCO_2 (µatm)	HCO_3^- (µlmol kg^{-1})	CO_3^{2} (µlmol kg^{-1})	Ω_{arag}
CT-COA	29.96 ± 0.023	8.10 ± 0.00417	2320.34 ± 9.75	448.07 ± 8.59	1789.07 ± 10.62	217.01 ± 2.06	3.53 ± 0.03
CT-OA	29.91 ± 0.038	7.90 ± 0.00021	2288.04 ± 14.42	886.65 ± 5.57	1943.42 ± 12.46	140.80 ± 1.00	2.29 ± 0.02
HT-COA	31.94 ± 0.035	8.16 ± 0.00380	2277.85 ± 13.29	386.63 ± 8.82	1653.63 ± 13.65	253.67 ± 2.96	4.18 ± 0.05
HT–OA	31.91 ± 0.042	7.90 ± 0.00012	2277.12 ± 14.68	883.61 ± 5.25	1913.74 ± 12.50	148.4 ± 1.07	2.44 ± 0.02

References Table S1:

S. Agostini, H. Fujimura, T. Higuchi, I. Yuyama, B. E. Casareto, Y. Suzuki, Y. Nakano, The effects of thermal and high-CO_2 stresses on the metabolism and surrounding microenvironment of the coral *Galaxea fascicularis*. *Comptes Rendus Biologies,* **336**, 384-391 (2013).

K. R. Anthony, D. I. Kline, G. Diaz-Pulido, S. Dove, O. Hoegh-Guldberg. Ocean acidification causes bleaching and productivity loss in coral reef builders. *Proceedings of the National Academy of Sciences of the United States of America,* **105**, 17442-17446 (2008).

G. Baghdasarian, A. Osberg, D. Mihora, H. M. Putnam, R. D. Gates, P. J. Edmunds Effects of temperature and pCO_2 on population regulation of *Symbiodinium* spp. in a tropical reef coral. *Biological Bulletin,* **232**, 123-139 (2017).
H. E. Bedwell-Ivers, M. S. Koch, K. E. Peach, L. Joles, E. Dutra, C. Manfrino, The role of *in hospite* zooxanthellae photophysiology and reef chemistry on elevated pCO_2 effects in two branching Caribbean corals: *Acropora cervicornis* and *Porites divaricata*. *ICES Journal of Marine Science,* **74**, 1103-1112 (2016).
E. F. Camp, D. J. Smith, C. Evenhuis, I. Enochs, D. Manzello, S. Woodcock, D. J. Suggett Acclimatization to high-variance habitats does not enhance physiological tolerance of two key Caribbean corals to future temperature and pH. *Proceedings: Biological sciences, The Royal Society,* **283**, (2016)
K. D. Castillo, J. B. Ries, J. F. Bruno, I. T. Westfield The reef-building coral *Siderastrea siderea* exhibits parabolic responses to ocean acidification and warming. *Proceedings: Biological sciences, The Royal Society,* **281**, (2014)
A. Chauvin, V. Denis, P. Cuet, Is the response of coral calcification to seawater acidification related to nutrient loading? *Coral Reefs,* **30**, 911-923 (2011).
S. Comeau, R. C. Carpenter, P. J. Edmunds, Coral reef calcifiers buffer their response to ocean acidification using both bicarbonate and carbonate. *Proceedings: Biological sciences, The Royal Society,* **280**, 20122374 (2012).
S. Comeau, R. C. Carpenter, P. J. Edmunds, Effects of irradiance on the response of the coral *Acropora pulchra* and the calcifying alga *Hydrolithon reinboldii* to temperature elevation and ocean acidification. *Journal of Experimental Marine Biology and Ecology,* **453**, 28-35 (2014a).
S. Comeau, R. C. Carpenter, P. J. Edmunds, Effects of pCO_2 on photosynthesis and respiration of tropical scleractinian corals and calcified algae. *ICES Journal of Marine Science,* **74**, 1092-1102 (2017).
S. Comeau, R. C. Carpenter, Y. Nojiri, H. M. Putnam, K. Sakai, P. J. Edmunds, Pacific-wide contrast highlights resistance of reef calcifiers to ocean acidification. *Proceedings of the Royal Society B: Biological Sciences,* **281**, 20141339-20141339 (2014b).
S. Comeau, C. E. Cornwall, T. M. DeCarlo, E. Krieger, M. T. McCulloch, Similar controls on calcification under ocean acidification across unrelated coral reef taxa. *Global Change Biology,* **24**, 4857-4868 (2018).
S. Comeau, C. E. Cornwall, C. A. Pupier, T. M. DeCarlo, C. Alessi, R. Trehern, M. T. McCulloch, Flow-driven micro-scale pH variability affects the physiology of corals and coralline algae under ocean acidification. *Scientific Reports,* **9**, (2019).
S. Comeau, P. J. Edmunds, N. B. Spindel, R. C. Carpenter, The responses of eight coral reef calcifiers to increasing partial pressure of CO_2 do not exhibit a tipping point. *Limnology and Oceanography,* **58**, 388-398 (2013).
S. Comeau, P. J. Edmunds, N. B. Spindel, R. C. Carpenter, Fast coral reef calcifiers are more sensitive to ocean acidification in short-term laboratory incubations. *Limnology and Oceanography,* **59**, 1081-1091 (2014c).
C. E. Cornwall, S. Comeau, T. M. DeCarlo, B. Moore, Q. D'Alexis, M. T. McCulloch, Resistance of corals and coralline algae to ocean acidification: physiological control of calcification under natural pH variability. *Proceedings of the Royal Society B: Biological Sciences,* **285**, 20181168 (2018).
A. Crawley, D. I. Kline, S. Dunn, K. R. N. Anthony, S. Dove, The effect of ocean acidification on symbiont photorespiration and productivity in *Acropora formosa*. *Global Change Biology,* **16**, 851-863 (2010).

S. W. Davies, A. Marchetti, J.B. Ries, K. D. Castillo, Thermal and *p*CO_2 stress elicit divergent transcriptomic responses in a resilient coral. *Frontiers in Marine Science,* **3**, 112 (2016).
G. Diaz-Pulido, M. Gouezo, B. Tilbrook, S. Dove, K. R. Anthony, High CO_2 enhances the competitive strength of seaweeds over corals. *Ecol Lett,* **14**, 156-162 (2011).
P. J. Edmunds, Zooplanktivory ameliorates the effects of ocean acidification on the reef coral *Porites* spp. *Limnology and Oceanography,* **56**, 2402-2410 (2011).
P. J. Edmunds, Effect of *p*CO_2 on the growth, respiration, and photophysiology of massive *Porites* spp. in Moorea, French Polynesia. *Marine Biology,* **159**, 2149-2160 (2012).
P. J. Edmunds, D. Brown, V. Moriarty, Interactive effects of ocean acidification and temperature on two scleractinian corals from Moorea, French Polynesia. *Global Change Biology,* **18**, 2173-2183 (2012).
I. C. Enochs, D. P. Manzello, R. Carlton, S. Schopmeyer, R. Hooidonk, D. Lirman, Effects of light and elevated *p*CO_2 on the growth and photochemical efficiency of *Acropora cervicornis*. *Coral Reefs*, **33**(2), 477-485 (2014).
K. E. Fabricius, C. Langdon, S. Uthicke *et al.,* Losers and winners in coral reefs acclimatized to elevated carbon dioxide concentrations. *Nature Climate Change,* **1**, 165-169 (2011).
M. Fine, D. Tchernov, Scleractinian coral species survive and recover from decalcification. *Science,* **315**, 1811-1811 (2007).
C. Godinot, F. Houlbreque, R. Grover, C. Ferrier-Pages, Coral uptake of inorganic phosphorus and nitrogen negatively affected by simultaneous changes in temperature and pH. *PLoS ONE,* **6**, e25024 (2011).
Y.-S. Hii, A. M. Ambok Bolong, T.-T. Yang, H.-C. Liew, Effect of elevated carbon dioxide on two scleractinian corals: *Porites cylindrica* (Dana, 1846) and *Galaxea fascicularis* (Linnaeus, 1767). *Journal of Marine Biology,* **2009**, 215196 (2009).
K. D. Hoadley, D. T. Pettay, D. Dodge, M. E. Warner, Contrasting physiological plasticity in response to environmental stress within different cnidarians and their respective symbionts. *Coral Reefs,* **35**, 529-542 (2016).
M. Holcomb, A. L. Cohen, D. C. McCorkle, An investigation of the calcification response of the scleractinian coral *Astrangia poculata* to elevated *p*CO_2 and the effects of nutrients, zooxanthellae and gender. *Biogeosciences,* **9**, 29-39 (2012).
M. Holcomb, D. C. McCorkle, A. L. Cohen, Long-term effects of nutrient and CO_2 enrichment on the temperate coral *Astrangia poculata* (Ellis and Solander, 1786). *Journal of Experimental Marine Biology and Ecology,* **386**, 27-33 (2010).
R. Horwitz, M. Fine, High CO_2 detrimentally affects tissue regeneration of Red Sea corals. *Coral Reefs*, **33**, 819–829 (2014).
F. Houlbrèque, R. Rodolfo-Metalpa, R. Jeffree *et al.,* Effects of increased *p*CO_2 on zinc uptake and calcification in the tropical coral *Stylophora pistillata*. *Coral Reefs,* **31**, 101-109 (2012).
A. Iguchi, S. Ozaki, T. Nakamura *et al.,* Effects of acidified seawater on coral calcification and symbiotic algae on the massive coral *Porites australiensis*. *Mar Environ Res,* **73**, 32-36 (2012).
P. L. Jokiel, K. S. Rodgers, I. B. Kuffner, A. J. Andersson, G. Cox, F. T. Meckenzie, Ocean acidification and calcifying reef organisms: a mesocosm investigation. *Coral Reefs,* **27**, 473-483 (2008).
C. P. Jury, R. F. Whitehead, A. M. Szmant, Effects of variations in carbonate chemistry on the calcification rates of *Madracis auretenra* (= *Madracis mirabilis* sensu Wells, 1973): bicarbonate concentrations best predict calcification rates. *Global Change Biology,* **16**, 1632-1644 (2010).

P. Kaniewska, P. R. Campbell, D. Kline, M. Rodriguez-Lanetty, D. J. Miller, S. Dove, O. Hoegh-Guldberg, Major cellular and physiological impacts of ocean acidification on a reef building coral. *PLoS ONE,* **7**, e34659 (2012).

J. Kavousi, J. D. Reimer, Y. Tanaka, T. Nakamura, Colony-specific investigations reveal highly variable responses among individual corals to ocean acidification and warming. *Marine Environmental Research,* **109**, 9-20 (2015).

S. Krief, E. J. Hendy, M. Fine, R. Yam, A. Meibom, G. L. Foster, A. Shemesh, Physiological and isotopic responses of scleractinian corals to ocean acidification. *Geochimica et Cosmochimica Acta,* **74**, 4988-5001 (2010).

T. Krueger, N. Horwitz, J. Bodin, M.-E. Giovani, S. Escrig, A. Meibom, M. Fine, Common reef-building coral in the Northern Red Sea resistant to elevated temperature and acidication. *R Soc Open Sci,* **4**, 170038 (2017).

C. Langdon, M. Aktinson, Effect of elevated pCO_2 on photosynthesis and calcification of corals and interactions with seasonal change in temperature/irradiance and nutrient enrichment. *Journal of Geophysical Research,* **110**, C09S07 (2005).

C. Langdon, R. Albright, A. Baker, P. Jones, Two threatened Caribbean coral species have contrasting responses to combined temperature and acidification stress. *Limnology and Oceanography,* **63**, 2450-2464 (2018).

N. Leclercq, J. P. Gattuso, J. Jaubert, Primary production, respiration, and calcification of a coral reef mesocosm under increased CO_2 partial pressure. *Limnology and Oceanography,* **47**, 558-564 (2002).

F. Marubini, M. J. Atkinson, Effects of lowered pH and elevated nitrate on coral calcification. *Marine Ecology Progress Series,* **188**, 117-121 (1999).

F. Marubini, H. Barnett, C. Langdon, M. J. Atkinson, Dependence of calcification on light and carbonate ion concentration for the hermatypic coral *Porites compressa*. *Marine Ecology Progress Series,* **220**, 153-162 (2001).

F. Marubini, C. Ferrier-Pagès, P. Furla, D. Allemand, Coral calcification responds to seawater acidification: a working hypothesis towards a physiological mechanism. *Coral Reefs,* **27**, 491-499 (2008).

F. Marubini, C. Ferrier-Pagès, J. P. Cuif, Suppression of skeletal growth in scleractinian corals by decreasing ambient carbonate-ion concentration: a cross-family comparison. *Proceedings of the Royal Society of London. Series B: Biological Sciences,* **270**, 179-184 (2003).

R. Z. B. Mason, Decline in symbiont densities of tropical and subtropical scleractinian corals under ocean acidification. Coral Reefs **37,** 945-953 (2018).

J. Movilla, E. Calvo, C. Pelejero, R. Coma, E. Serrano, P. Fernández-Vallejo, M. Ribes, Calcification reduction and recovery in native and non-native Mediterranean corals in response to ocean acidification. *Journal of Experimental Marine Biology and Ecology,* **438**, 144-153 (2012).

N. Muehllehner, P. E. Edmunds, Effects of ocean acidification and increased temperature on skeletal growth of two scleractinian corals, *Pocillopora meandrina* and *Porites rus*. In: *11th International Coral Reef Symposium.*, (Ft. Lauderdale, Florida, 2008) pp 57-61

S. H. C. Noonan, K. E. Fabricius, Ocean acidification affects productivity but not the severity of thermal bleaching in some tropical corals. *ICES Journal of Marine Science: Journal du Conseil*, fsv127, (2015).

D. Ogawa, T. Bobeszko, T. Ainsworth, W. Leggat, The combined effects of temperature and CO_2 lead to altered gene expression in *Acropora aspera*. *Coral Reefs,* **32**, 895-907 (2013).

S. Ohde, M. M. M. Hossain, Effect of $CaCO_3$ (aragonite) saturation state of seawater on calcification of *Porites* coral. *Geochemical Journal,* **38**, 613-621 (2004).
S. Ohki, T. Irie, M. Inoue *et al.,* Symbiosis increases coral tolerance to ocean acidification. *Biogeosciences Discussions,* **10**, 7013-7030 (2013).
R. R. Okazaki, P. K. Swart, C. Langdon, Stress-tolerant corals of Florida Bay are vulnerable to ocean acidification. *Coral Reefs,* **32**, 671-683 (2013).
D. A. Renegar, B. Riegl, Effect of nutrient enrichment and elevated CO_2 partial pressure on growth rate of Atlantic scleractinian coral *Acropora cervicornis*. *Marine Ecology Progress Series,* **293**, 69-76 (2005).
S. Reynaud, N. Leclercq, S. Romaine-Lioud, C. Ferrier-Pagès, J. Jaubert, J. P. Gattuso, Interacting effects of CO_2 partial pressure and temperature on photosynthesis and calcification in a scleractinian coral. *Global Change Biology,* **9**, 1660-1668 (2003).
J. B. Ries, A. L. Cohen, D. C. McCorkle, Marine calcifiers exhibit mixed responses to CO_2-induced ocean acidification. *Geology,* **37**, 1131-1134 (2009).
J. B. Ries, A. L. Cohen, D. C. McCorkle, A nonlinear calcification response to CO_2-induced ocean acidification by the coral *Oculina arbuscula*. *Coral Reefs,* **29**, 661-674 (2010).
R. Rodolfo-Metalpa, F. Houlbrèque, É. Tambutté *et al.,* Coral and mollusc resistance to ocean acidification adversely affected by warming. *Nature Climate Change,* **1**, 308-312 (2011).
R. Rodolfo-Metalpa, S. Martin, C. Ferrier-Pagès, J. P. Gattuso, Response of the temperate coral *Cladocora caespitosa* to mid- and long-term exposure to pCO_2 and temperature levels projected for the year 2100 AD. *Biogeosciences,* **7**, 289-300 (2010).
V. Schoepf, A. G. Grottoli, M. E. Warner *et al.,* Coral energy reserves and calcification in a high-CO_2 world at two temperatures. *PLoS ONE,* **8**, e75049 (2013).
D. J. Suggett, L. F. Dong, T. Lawson, E. Lawrenz, L. Torres, D. J. Smith, Light availability determines susceptibility of reef building corals to ocean acidification. *Coral Reefs,* **32**, 327-337 (2013).
D. J. Suggett, J. M. Hall-Spencer, R. Rodolfo-Metalpa *et al.,* Sea anemones may thrive in a high CO_2 world. *Global Change Biology,* **18**, 3015-3025 (2012).
A. Takahashi, H. Kurihara, Ocean acidification does not affect the physiology of the tropical coral *Acropora digitifera* during a 5-week experiment. *Coral Reefs,* **32**, 305-314 (2013).
T. Towanda, E. V. Thuesen, Prolonged exposure to elevated CO_2 promotes growth of the algal symbiont *Symbiodinium muscatinei* in the intertidal sea anemone *Anthopleura elegantissima*. *Biol Open,* **1**, 615-621 (2012).
P. Tremblay, M. Fine, J. F. Maguer, R. Grover, C. Ferrier-Pagès, Photosynthate translocation increases in response to low seawater pH in a coral–dinoflagellate symbiosis. *Biogeosciences,* **10**, 3997-4007 (2013).
A. A. Venn, E. Tambutté, M. Holcomb, J. Laurent, D. Allemand, S. Tambutté, Impact of seawater acidification on pH at the tissue–skeleton interface and calcification in reef corals. *Proceedings of the National Academy of Sciences,* **110**, 1634-1639 (2013).
C. B. Wall, T. Y. Fan, P. J. Edmunds, Ocean acidification has no effect on thermal bleaching in the coral *Seriatopora caliendrum*. *Coral Reefs,* **33**, 119-130 (2014).

C. B. Wall, R. A. B. Mason, W. R. Ellis, R. Cunning, R. D. Gates, Elevated pCO_2 affects tissue biomass composition, but not calcification, in a reef coral under two light regimes. *Royal Society Open Science,* 4, 170683 (2017).
