# Supplementary material for: Evaluation of the current understanding of the impact of climate change on coral physiology after three decades of experimental research
Source: Commun Biol. 2022 Dec 26;5:1418. doi: 10.1038/s42003-022-04353-1 (PMC9792581; doi:10.1038/s42003-022-04353-1)
Supplement: Supplementary file 5 — Reporting Summary [file 42003_2022_4353_MOESM5_ESM.pdf]

nature portfolio

Corresponding author(s): Susana Enríquez

Last updated by author(s): 2022/10/5

Reporting Summary

Nature Portfolio wishes to improve the reproducibility of the work that we publish. This form provides structure for consistency and transparency in reporting. For further information on Nature Portfolio policies, see our Editorial Policies and the Editorial Policy Checklist.

Statistics

For all statistical analyses, confirm that the following items are present in the figure legend, table legend, main text, or Methods section.

n/a	Confirmed	
☐	☒	The exact sample size (*n*) for each experimental group/condition, given as a discrete number and unit of measurement
☐	☒	A statement on whether measurements were taken from distinct samples or whether the same sample was measured repeatedly
☐	☒	The statistical test(s) used AND whether they are one- or two-sided<br>*Only common tests should be described solely by name; describe more complex techniques in the Methods section.*
☐	☒	A description of all covariates tested
☐	☒	A description of any assumptions or corrections, such as tests of normality and adjustment for multiple comparisons
☐	☒	A full description of the statistical parameters including central tendency (e.g. means) or other basic estimates (e.g. regression coefficient) AND variation (e.g. standard deviation) or associated estimates of uncertainty (e.g. confidence intervals)
☐	☒	For null hypothesis testing, the test statistic (e.g. *F*, *t*, *r*) with confidence intervals, effect sizes, degrees of freedom and *P* value noted<br>*Give P values as exact values whenever suitable.*
☒	☐	For Bayesian analysis, information on the choice of priors and Markov chain Monte Carlo settings
☒	☐	For hierarchical and complex designs, identification of the appropriate level for tests and full reporting of outcomes
☒	☐	Estimates of effect sizes (e.g. Cohen's *d*, Pearson's *r*), indicating how they were calculated

Our web collection on statistics for biologists contains articles on many of the points above.

Software and code

Policy information about availability of computer code

Data collection	We used the manufacturer's software (USB2000+ and Spectrasuite, Ocean Optics, USA) for the control of the miniature spectrometer in light absorption and calcification determintations. To calculate the components of the carbonate system in seawater (pCO2, CO32-, HCO3-, DIC concentrations and Ωarag), we used the CO2SYS software in Microsoft Excel, according to Pierrot, D., Lewis, E. & Wallace, D. W. R. MS Excel Program Developed for CO2 System Calculations, 2006). The seawater carbonate system of the OA treatments was automatically adjusted by bubbling with CO2 using electronic valves (Sierra Instruments, INC, Smart-Track 2) until reaching the desired pH. Water temperature and pH were continuously monitored using a pH-electrode (resolution of 0.01 pH units; Thermo Scientific, Inc., USA) and a thermocouple-based custom-made probes (J-types; resolution of 0.1°C; TEI-Ingeniería, Mexico), respectively, both connected to a computer-based system equipped with a wireless data acquisition card (National Instruments, Texas, USA).
Data analysis	Validation of AT values was performed using certified reference material from the Andrew Dickson's lab (Scripps Institution of Oceanography, USA). We used SPSS Statistics 20.0 (IBM Inc., USA) for the data analyses,

For manuscripts utilizing custom algorithms or software that are central to the research but not yet described in published literature, software must be made available to editors and reviewers. We strongly encourage code deposition in a community repository (e.g. GitHub). See the Nature Portfolio guidelines for submitting code & software for further information.

Data

Policy information about availability of data

All manuscripts must include a data availability statement. This statement should provide the following information, where applicable:

- Accession codes, unique identifiers, or web links for publicly available datasets
- A description of any restrictions on data availability
- For clinical datasets or third party data, please ensure that the statement adheres to our policy

All data are available in the main text or the supplementary materials.

Human research participants

Policy information about studies involving human research participants and Sex and Gender in Research.

Reporting on sex and gender	No human research was performed
Population characteristics	*Describe the covariate-relevant population characteristics of the human research participants (e.g. age, genotypic information, past and current diagnosis and treatment categories). If you filled out the behavioural & social sciences study design questions and have nothing to add here, write "See above."*
Recruitment	*Describe how participants were recruited. Outline any potential self-selection bias or other biases that may be present and how these are likely to impact results.*
Ethics oversight	*Identify the organization(s) that approved the study protocol.*

Note that full information on the approval of the study protocol must also be provided in the manuscript.

Field-specific reporting

Please select the one below that is the best fit for your research. If you are not sure, read the appropriate sections before making your selection.

☒ Life sciences ☐ Behavioural & social sciences ☐ Ecological, evolutionary & environmental sciences

For a reference copy of the document with all sections, see nature.com/documents/nr-reporting-summary-flat.pdf

Life sciences study design

All studies must disclose on these points even when the disclosure is negative.

Sample size	We used four replicates of each of the four species investigated for the characterization of each parameter. Corals were characterized at day 0 (initials) and after 10 days of exposure to four different treatments (control, heat stress, ocean acidification-OA, and combined effect of heat stress and OA). Samples were exposed to experimental conditions in 8 tanks (n = 2 per treatment, 2 coral nubbins per species and tank).The average flow rate in the experimental tanks was 0.33 L s−1 with a turnover rate of about 90 s, which allowed resolving the problem of the low number of tank replicates. Increasing this number was not possible for the complexity of this comparative analysis (four species under four different treatments). Experimental corals were fragments obtained from three different colonies for each of species analyzed.
Data exclusions	No data were excluded from the analysis
Replication	To test the effects of heat-stress and OA, we used a fully orthogonal, two-factor design. Corals were maintained in a water table with a seawater flow-through system, with a fast turnover rate of about 90 s (average flow rate 0.33 L s−1). Experimental 30-L tanks were supplied with constant seawater from four 1000-L header tanks, which had access to direct seawater flow from the reef lagoon. Samples were collected at similar depth (4–5 m) in the back-reef of Puerto Morelos, Mexico. After collection, coral fragments were immediately transferred to our mesocosm system located nearby the reef lagoon. Fragments were placed in outdoor tanks with running seawater from the lagoon. All organisms were kept under natural solar illumination dampened to 47% of surface irradiance (Es) levels using neutral screening,. This irradiance corresponded to the light intensity at the sampling depth. One day after sampling, corals were cut into smaller pieces of roughly equal size (2 cm2). Experimental organisms were allowed to recover for two days before gluing them onto PVC plates. After this treatment, coral nubbins were transferred back to the reef lagoon for full recovery, where they were fixed onto tables placed at 4 m depth in the back reef. The nubbins used in the experiment described in this manuscript were maintained in the reef lagoon of Puerto Morelos for 1 year before the experiment was performed in August-September 2013. The temperature treatments were set based on the local summer maximum of the reef lagoon of Puerto Morelos (30°C) achieved in August (ambient control temperature– CT), and we used a thermal anomaly of +2 °C above this summer average (32°C) as the heat-stress treatment (HT). Water temperature was independently regulated using titanium immersion heaters (Process Technology, Ohio, USA) in the main reservoirs, which supplied seawater to the experimental tanks. For the OA-treatments, the current seawater pH in the lagoon (pH 8.1) was used as a control condition (ambient pH – COA) and the changes expected by 2100 under the carbon emission scenario B142 or SSP2-4.543, with pH units in the National Bureau of Standards (NBS) scale of pH=7.9, were used to determine the OA-treatment (OA). The seawater carbonate system of the OA treatments was automatically adjusted by bubbling with CO2 using electronic valves (Sierra Instruments, INC, Smart-Track 2) until reaching the desired pH. Well-mixed water was continuously

pumped from the main reservoir to the corresponding experimental tanks. Water temperature and pH were continuously monitored using a pH-electrode (resolution of 0.01 pH units; Thermo Scientific, Inc., USA) and a thermocouple-based custom-made probes (J-types; resolution of 0.1°C; TEI-Ingeniería, Mexico), respectively, both connected to a computer-based system equipped with a wireless data acquisition card (National Instruments, Texas, USA). Throughout the experiment, we used natural solar irradiance and neutral screening to maintain the pre-acclimatory conditions (47% Es). Variation in solar irradiance was monitored using a cosine-corrected sensor LI-192 quantum sensor (LI-COR, Lincoln, USA) connected to a data logger (LI-COR LI-1400, Lincoln, USA) and registered in 5-minute intervals at the pier of the UASA. Due to the time required to perform the physiological determinations of four species, each treatment was initiated with a delay of one day in a progressive manner. This allowed finishing all physiological determinations of one treatment in one day, while maintaining the same duration of each treatment for all organisms. The experiment was started with the control treatment (CT-COA), followed by the CT-OA treatment on day 1, the HT-COA on day 2 and ultimately, HT-OA on day 3

Randomization	Experimental corals were located randomly in the tanks, 2 samples per treatment and species in each of the 8 tanks (2 coral nubbins per species and tank), to a total of 4 replicates characterized for each species (4 spp) and treatment (4 treatments). We homogenized light and flow conditions within each tank and among tank to minimize the environmental variability within tanks.
Blinding	Physiological characterizations are time consuming and limit the number of replicates that can be determined at a specific time. In this comparative analysis we analysed the response of four species to four different experimental treatments. In order to account for part of the natural variability of the local coral population we collected four different colonies per species located at the same depth, and in order to buffer the intracolony variability in photoacclimation, we let the coral nubbins to fully recover and acclimatize to similar environmental conditions and incident light (no variation in the slope of the tissue) in the reef lagoon for one year, and when all samples were fully acclimatized to the local summer maximum of temperature (30ºC), we transferred the samples to the experimental tanks. So, we used a homogeneous experimental population of coral nubbins already acclimatize to the local peak of temperature in summer. The position of the samples in the tanks was random. The only attention we paid for the selection of the nubbins was to make sure that we were selecting samples from the three different colonies collected for each species, at time 0 of the experiment and at time 10 in each of the four treatments.

Reporting for specific materials, systems and methods

We require information from authors about some types of materials, experimental systems and methods used in many studies. Here, indicate whether each material, system or method listed is relevant to your study. If you are not sure if a list item applies to your research, read the appropriate section before selecting a response.

Materials & experimental systems

n/a	Involved in the study
☒	☐ Antibodies
☒	☐ Eukaryotic cell lines
☒	☐ Palaeontology and archaeology
☐	☒ Animals and other organisms
☒	☐ Clinical data
☒	☐ Dual use research of concern

Methods

n/a	Involved in the study
☒	☐ ChIP-seq
☒	☐ Flow cytometry
☒	☐ MRI-based neuroimaging

Animals and other research organisms

Policy information about studies involving animals; ARRIVE guidelines recommended for reporting animal research, and Sex and Gender in Research

Laboratory animals	Our study did not involve laboratory animals. The animals studied were symbiotic corals (no vertebrates or higher invertebrates were studied).
Wild animals	Coral fragments of three colonies of Pseudodiploria strigosa, Montastraea cavernosa, Orbicella annularis and Orbicella faveolata, were collected in the back-reef of Puerto Morelos, Mexico (20°54'19.80"N, 86°50'6.20"W), and immediately transferred to the mesocosm system of our department (UASA_UNAM) and located in outdoor tanks with running seawater from the lagoon and light conditions similar at the sampling depth. One day after sampling, corals were cut into smaller pieces of roughly equal size (2 cm2) and allowed to recover for two days before gluing them onto PVC plates. Then, these organisms were transferred back to the reef lagoon for full recovery, and fixed onto tables placed at 4 m depth. The nubbins used in this experiment were maintained in the reef lagoon of Puerto Morelos for 1 year before the experiment was performed in August-September 2013.
Reporting on sex	Sex was not considered in this analysis as the sexual effort of symbiotic corals and their capacity to produce germ cells is absent when the colony fragments are reduced in size (=2 cm2). This is why these experimental corals are usually named "nubbins".
Field-collected samples	Coral fragments kept for a year on tables located at the back reef of the lagoon, were transferred back to the UNAM mesocosm facilities of Puerto Morelos. The temperature in the control tanks (CT) was 29.93 ± 0.19°C and corresponded to the local maximum mean in summer in August (cf. Scheufen et al 2017), collecand the temperature of acclimatization of the experimental organisms “in situ”. The high temperature treatment (HT; average of 31.93 ± 0.25 °C)22, represented +2ºC above the local summer maximum. Seawater chemistry in the experimental tanks showed aragonite saturation state values (Ωarag) ~40% lower (Ωarag = 2.29–2.44) in the OA treatment (OA; pH=7.9) than at ambient control-pH (COA; pH=8.1; Ωarag=3.53–4.18). The concentration of HCO3- increased from ~1653 mol kg-1 at pH=8.1 to 1943 mol kg-1 at pH=7.9, and CO2 concentrations increased from ~387 to 887 µatm. In contrast, the average CO32- concentration decreased from 217–253 µmol kg-1 in the control/ambient treatment to 140-148 µmol kg-1 in the

OA, pH=7.9, treatment. Total alkalinity was not affected by the reduction in seawater pH, remaining within the range of 2277–2320 (µmol kg-1). Natural variation in solar irradiance during the experiment presented an average in diurnal light exposure of 14.1 ± 1.14 mol quanta m-2 d-1. This variability simulated the natural light conditions to which organisms were pre-acclimated and recovered "in situ" in the reef lagoon of Puerto Morelos after their manipulation. From day 6 until day 10 of the experiment, we measured a 60% reduction in light exposure due to very dense cloud cover (Fig.1a).
The temperature treatments were set based on the local summer maximum of the reef lagoon of Puerto Morelos (30°C)5 achieved in August (ambient control temperature– CT), and a thermal anomaly of +2 °C above this summer average (32°C) considered the heat-stress treatment (HT). Water temperature was independently regulated using titanium immersion heaters (Process Technology, Ohio, USA) in the main reservoirs, which supplied seawater to the experimental tanks. For the OA-treatments, the current seawater pH in the lagoon (pH 8.1) was used as a control condition (ambient pH – COA) and the changes expected by 2100 under the carbon emission scenario B142 or SSP2-4.543, with pH units in the National Bureau of Standards (NBS) scale of pH=7.9, were used to determine the OA-treatment (OA). The seawater carbonate system of the OA treatments was automatically adjusted by bubbling with CO2 using electronic valves (Sierra Instruments, INC, Smart-Track 2) until reaching the desired pH. Well-mixed water was continuously pumped from the main reservoir to the corresponding experimental tanks. Water temperature and pH were continuously monitored using a pH-electrode (resolution of 0.01 pH units; Thermo Scientific, Inc., USA) and a thermocouple-based custom-made probes (J-types; resolution of 0.1°C; TEI-Ingeniería, Mexico), respectively, both connected to a computer-based system equipped with a wireless data acquisition card (National Instruments, Texas, USA). Throughout the experiment, we used natural solar irradiance and neutral screening to maintain the pre-acclimatory conditions (47% Es). Variation in solar irradiance was monitored using a cosine-corrected sensor LI-192 quantum sensor (LI-COR, Lincoln, USA) connected to a data logger (LI-COR LI-1400, Lincoln, USA) and registered in 5-minute intervals at the pier of the UASA. Due to the time required to perform the physiological determinations of four species, each treatment was initiated with a delay of one day in a progressive manner. This allowed finishing all physiological determinations of one treatment in one day, while maintaining the same duration of each treatment for all organisms. The experiment was started with the control treatment (CT-COA), followed by the CT-OA treatment on day 1, the HT-COA on day 2 and ultimately, HT-OA on day 3.

Ethics oversight

The coral sampling was performed under the Mexican permit 'Permiso de Pesca de Fomento no. DGOPA.08606.251011.3021' provided by the Secretaría de Agricultura, Ganadería, Desarrollo rural, Pesca y Alimentación, of the Comisión Nacional de Acuacultura y Pesca of the Dirección General de Ordenamiento Pesquero y Acuícola (DGOPA) of the United States of Mexico.

Note that full information on the approval of the study protocol must also be provided in the manuscript.
